# Applications of Surface Modification Technologies in Nanomedicine for Deep Tumor Penetration

**DOI:** 10.1002/advs.202002589

**Published:** 2020-11-27

**Authors:** Zimu Li, Xiaoting Shan, Zhidong Chen, Nansha Gao, Wenfeng Zeng, Xiaowei Zeng, Lin Mei

**Affiliations:** ^1^ Institute of Pharmaceutics School of Pharmaceutical Sciences (Shenzhen) Sun Yat‐sen University Shenzhen 518107 China; ^2^ Tianjin Key Laboratory of Biomedical Materials Key Laboratory of Biomaterials and Nanotechnology for Cancer Immunotherapy Institute of Biomedical Engineering Chinese Academy of Medical Sciences and Peking Union Medical College Tianjin 300192 China

**Keywords:** cancer therapy, penetration evaluation, solid tumors, surface modifications, tumor penetration

## Abstract

The impermeable barrier of solid tumors due to the complexity of their components limits the treatment effect of nanomedicine and hinders its clinical translation. Several methods are available to increase the penetrability of nanomedicine, yet they are too complex to be effective, operational, or practical. Surface modification employs the characteristics of direct contact between multiphase surfaces to achieve the most direct and efficient penetration of solid tumors. Furthermore, their simple operation makes their use feasible. In this review, the latest surface modification strategies for the penetration of nanomedicine into solid tumors are summarized and classified into “bulldozer strategies” and “mouse strategies.” Additionally, the evaluation methods, existing problems, and the development prospects of these technologies are discussed.

## Graphical abstract

This review provides a timely and comprehensive summary and discussion of the existing penetration‐promoting surface modification strategies and possible future development directions based on the tumor penetration dilemma caused by the interaction of components in the tumor microenvironment. In addition, the evaluation methods of nanomedicine penetrability are summarized, and the challenges of surface modification for tumor penetration are discussed.

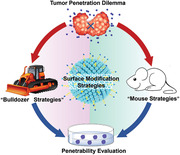

## 1 Introduction

A human solid tumor is a complex combination of tumor cells, tumor blood vessels, extracellular matrix, and metabolic waste difficult to penetrate by ordinary nanomedicine.^[^ ^1^ ^]^ Despite the fact that various surface modification strategies have achieved long‐term circulation of nanomedicine in vivo^[^ ^2^ ^]^ and good enrichment in the vicinity of solid tumors through active or passive targeting strategies,^[^ ^3^ ^]^ the therapeutic effect of these drugs remains limited by the inability to penetrate deep into solid tumors.^[^ ^4^ ^]^ Thus, the impermeability of human solid tumors is one of the key factors hindering the clinical application of nanomedicine. Furthermore, rapidly induced solid tumors in immunodeficient mice are commonly used as animal models of solid tumor research, yet these cannot realistically simulate human solid tumors since animal tumors require little penetrability of nanomedicine to produce good therapeutic results, thus explaining the fact that nanomedicines that can penetrate animal tumors are less effective in humans.^[^ ^5^ ^]^

Cancer stem cells are root cells that maintain the vitality of the tumor cell population and play a crucial role in the occurrence, proliferation, metastasis, and recurrence of solid tumors.^[^ ^6^ ^]^ Several studies have shown that cancer stem cells are one of the greatest obstacles to eradicating tumors.^[^ ^7^ ^]^ These cells are not only rare in number but can also hide in the depths of tumors that lack oxygen and blood vessels, preventing most chemotherapy drugs and nanocarriers from getting close enough to kill them.^[^ ^8^ ^]^ Therefore, there is an urgent need to study the penetrability of nanomedicine in solid tumors.

Surface modification leads to the endowment of new properties and functions, such as hydrophilicity/hydrophobicity, surface charge properties, biocompatibility, roughness, adhesion, or optical and magnetic properties, to the surface of materials whilst retaining their original bulk properties.^[^ ^9^ ^]^ As a common method of nanomedicine modification, surface modification has been widely studied in recent years,^[^ ^10^ ^]^ and advanced and practical surface modification strategies emerge in an endless stream,^[^ ^11^ ^]^ thus expanding the scope of application of nanomedicine. Generally speaking, in the process of delivery, the first and most direct interactions with the tumor microenvironment involve those between the tumor and the surface of nanomedicine, and such interactions will directly determining the penetration effect of nanomedicine into solid tumors. The surface modification of most nanomedicines can be performed efficiently using mild and easily achievable conditions,^[^ ^12^ ^]^ making the large‐scale production and clinical application feasible. Thus, surface modification is one of the most simple and efficient modification methods to achieve deep penetration of solid tumors.

**Figure 1:**
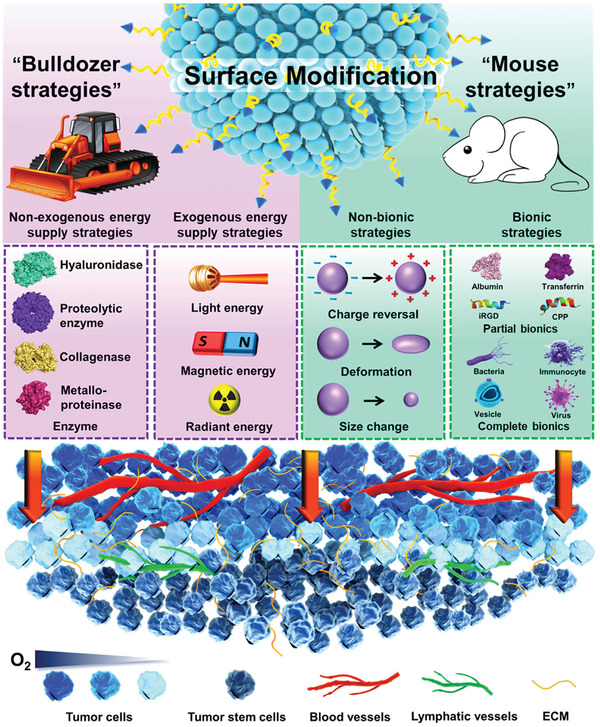
Schematic illustration of "bulldozer strategies" and "mouse strategies" of surface modification for nanomedicine deep penetration.

Many previous strategies to promote the penetration of nanomedicine are complex, resulting in complex preparation processes and low yields, which are extremely detrimental to the realization of large‐scale production and clinical application. Targeting the trickiest problem of solid tumor deep penetration of nanomedicine, the thinking of effective surface modification was chosen as the breakthrough point, hoping to provide the reference for scientific researchers and promote contribution to resolve the problem. This review discusses the composition of a solid tumor microenvironment to analyze the multiple causes of penetration difficulty and classifies the available surface modification strategies to promote solid tumor penetration into two types, termed “bulldozer strategies” and “mouse strategies” (**Figure** **1**). Finally, the existing methods to assess the penetrability of nanomedicines are summarized.

## 2 Composition of Solid Tumors and Obstruction of Nanomedicine Penetration

Jain et al.^[^ ^13^ ^]^ showed that solid tumors are not just a collection of malignant mutated cells but are rather composed of a tumor microenvironment comprised of cancer cells, blood, and lymphatic vessels as well as a variety of non‐malignant host cells and metabolic waste. In this way, the rapid growth and migration of solid tumors depend not only on tumor cells with gene mutations but also on cells and secretions providing favorable conditions for tumor growth. Indeed, most current therapies for solid tumors target not only to tumor cells but also to the tumor microenvironment, leading to the improved efficacy and success rates of antitumor therapies.^[^ ^14^ ^]^

Besides their complexity, the biochemical and physical microenvironments of tumors are also highly dynamic, and vary with tumor growth and cell migration.^[^ ^15^ ^]^ Thus, acting as a “strong and complex fortress,” solid tumors are highly resistant to the deep penetration of nanomedicine. Therefore, the review analyzes the microenvironment composition of solid tumor and summarizes the reasons that hinder the deep penetration of nanomedicine, laying a foundation for finding more reliable penetration promotion strategies.

### 2.1 Abnormal Blood Vessels

**Figure 2:**
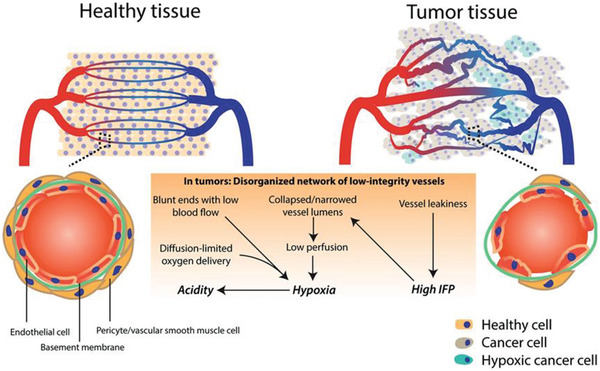
The differences between vasculature in normal tissue and tumor. Reproduced with permission.^[^ ^17^ ^]^ Copyright 2018, Springer Nature.

Most antitumor drugs and nanomedicine are delivered by blood, highlighting the need to properly assess the nature of blood vessels in solid tumors when researching the deep penetration of nanomedicine.^[^ ^16^ ^]^ Normal vessels are orderly, whereas abnormal vasculature in the solid tumor is quite tortuous, irregular, and chaotic.^[^ ^17^ ^]^ By contrast, there is nearly no orderly structure from large vessels to sequential and smaller vessels in solid tumors (**Figure** **2**).

The formation of a tumor vascular network includes the formation of new vessels from established vascular beds through the differentiation of endothelial precursors from bone marrow or by the co‐option and modification of existing vessels, resulting in a heterogeneous tumor vasculature.^[^ ^17^ ^]^ Such heterogeneity will lead to vascular distortion, vascular endothelial integrity, and other structural abnormalities, affecting blood flow and the distribution that play key roles in nanomedicine penetration. This abnormality in tumor vessels leads to many obstacles in drug delivery. First, the heterogeneity of the tumor vasculature will lead to poor penetration of nanomedicine. Second, the low density of vessels in deep tumors leads to poor penetration as it is difficult for nanomedicine to be transported into deep tumors without adequate blood flow and regular vascular distribution.^[^ ^18^ ^]^ Additionally, in solid tumors, hypoxia and acidosis lead to a higher viscous blood flow,^[^ ^19^ ^]^ further hindering the penetration ability of nanomedicine.

The poor penetration of nanomedicine is also caused by the difference in the enhanced permeability and retention (EPR) effect between animal experimental tumor models and human tumors. Various studies have shown that vascular permeability and hydraulic conductivity in tumors are higher than in normal tissue,^[^ ^20^ ^]^ forming the basis of the EPR effect.^[^ ^21^ ^]^ Not all vessels in the tumor are excessively leaky due to the heterogeneity of tumor vasculature, which means that the effect of the EPR effect cannot be overstated.^[^ ^21^ ^]^ As a result, many data and conclusions from animal models cannot be replicated in patients because of the poor penetration of nanomedicine. It follows that the deep penetration of the tumor should be achieved through the modification of nanocarriers instead of the EPR effect, among which surface modification is the most simple and efficient one.

### 2.2 Abnormal Lymphatic Vessels

Poor nanomedicine penetration is also caused by the existence of abnormal lymphatic vessels in solid tumors. Like vasculature, lymphatic vessels in solid tumors are also abnormal in both structure and function.^[^ ^22^ ^]^ In normal tissue, the lymphatic network discharges excess fluid that arises mainly because of the metabolism from tissue, which can balance tissue interstitial fluid and maintain ideal tissue function.^[^ ^23^ ^]^ In solid tumors, proliferating tumor cells and stromal cells will cause the collapse of lymphatic vessels,^[^ ^24^ ^]^ such that fluid cannot be removed from the tumor, causing edema and the increase of interstitial fluid pressure (IFP),^[^ ^22^ ^]^ which contribute to the poor penetration of nanomedicine. Lymphatic vessels with practical function only exist in the periphery of solid tumors and are likely to remove nanomedicine that has not been transported into the solid tumor, thus largely influencing the penetration capacity of nanomedicine.

### 2.3 Abnormal Extracellular Matrix (ECM)

Similar to other components in solid tumors, considerable differences are observed between the ECM in tumors and that in normal tissues. The ECM has a complex and dynamic composition that leads to high solid and liquid pressure, affecting the penetration of nanomedicine.

The IFP will adversely affect the penetration of nanomedicine into solid tumors. In normal tissue, the value of IFP arranges from 0 to 3 mm Hg, whereas that in solid tumors ranges from 5 to 40 mm Hg.^[^ ^25^ ^]^ The solid pressure from tumor cells not only deform vasculature but also result in interstitial hypertension, a combo of invalid drainage of fluid from the tumor center and leakage of tumor vessels, which further influence the penetration of nanomedicine through the high IFP.^[^ ^26^ ^]^ Provenzan et al. found that, through the exposure of enzymatic of hyaluronan, a decrease in density of the ECM and IFP can be observed,^[^ ^27^ ^]^ showing that the ECM is an important factor contributing to a high IFP in solid tumors and that a low IFP will contribute to the penetration of nanomedicine.

The solid pressure of the ECM embodies specifically in density and stiffness, which will create the physical barrier, preventing deep penetration of nanomedicine and further increase IFP.^[^ ^28^ ^]^ Proliferating tumor cells and stromal cells will cause excessive production of tumor ECM and excessive stiffness of tumor ECM (compared to normal tissue) can be due to desmoplasia as well as ECM reorganization and crosslinking.^[^ ^29^ ^]^ The tumor ECM includes components such as proteins, glycoproteins, proteoglycans, and polysaccharides, largely influencing the properties of the ECM.^[^ ^30^ ^]^ Together with abnormal vasculature, a dense ECM will further increase the IFP and thus affect nanomedicine penetration.

The ECM is highly dynamic. Abnormal changes in the amount and composition of the ECM have been observed, including a change of various collagens, including collagen I, II, III, V, and IX, matrix metalloproteinases (MMP) activity, and many other ECM components and their receptors.^[^ ^31^ ^]^ Changes in remodeling enzymes, such as crosslinking enzymes of the lysyl oxidase family and cathepsins are also observed.^[^ ^32^ ^]^ These remodeling enzymes can change the components and their proportion of solid tumors directly. Thus, these changes in ECM dynamics may result from one or more changes in its components, leading to major challenges in the tailoring of nanomedicine penetration and drug delivery due to its uncertainty and complexity.

### 2.4 Tumor Cells

Tumor cells and normal cells have many intrinsic differences, with the greatest difference being the abnormal speed of growth of tumor cells. Tumor cells can grow indefinitely, resulting in an extremely large quantity of tumor cells in solid tumors. Under the combined action of a dense ECM, proliferating tumor cells will contribute to the density of solid tumors and form a physical barrier that results in poor penetration.

### 2.5 Non‐Malignant Host Cells

In addition to tumor cells and their secretions, host stromal cells, consisting of endothelial cells, fibroblasts, and various inflammatory and immune cells, are also crucial components in solid tumors and will affect nanomedicine penetration.

Tumor‐associated fibroblast cells (TAFs), a kind of spindle‐shaped cell, can be found in almost every solid tumor. As the crucial components in the tumor microenvironment, TAFs mediate ECM remodeling, enhance cancer cell proliferation, contribute to immune suppression, and even in connection with anti‐cancer drug resistance.^[^ ^33^ ^]^ Thus, the penetration of nanomedicine within the tumor may be closely dependent on TAFs.^[^ ^1a^ ^]^ TAFs principally aggregate in the vicinity of tumor vessels, so that a large number of nanoparticles will first be attracted to TAFs. A sevenfold increase in nanoparticle assimilation can be seen in TAFs at 16 h post‐intravenous injection, which means that only a small portion of nanomedicine can be available in penetrating into the deeper tumor. Furthermore, TAFs exhibit an abnormal growth speed in relation to normal fibroblasts, which will further increase the density of solid tumors and harden the physical barrier, leading to difficulties for nanomedicine penetration.

Tumor‐associated macrophages (TAMs) are another kind of common component in the solid tumor microenvironment and play significant roles in immunosuppression and in the formation of the tumor inflammatory microenvironment.^[^ ^14^, ^34^ ^]^ Similar to TAFs, TAMs also contribute to the poor penetration of nanoparticles due to their phagocytic ability, leading to the accumulation of nanomedicine and thus to decreased nanomedicine deep tumor penetration.^[^ ^25^ ^]^ Additionally, due to the immunosuppression conferred by TAMs, the tumor will grow rapidly, leading to the formation of dense solid tumors and hard physical barriers, resulting in poor nanomedicine penetration.

## 3 “Bulldozer Strategies” of Surface Modification for Nanomedicine Deep Penetration

Bulldozer, as a commonly used obstacle remover, always does a good job in road work, following the strategy of confronting the toughness with toughness. Inspired by this, in order to break through the solid tumor fortress and realize deep penetration, many nanocarriers can achieve the function like a bulldozer through surface functionalization. With the delivery of nanomedicine, the disruption of solid tumor defense can be achieved through the destruction of the tumor microenvironment, such that the deep penetration of nanomedicine can be promoted. “Bulldozer strategies” of surface modification can be divided into two types according to whether or not an exogenous energy supply is needed.

### 3.1 Surface Modification Strategies without Exogenous Energy Supply

**Figure 3:**
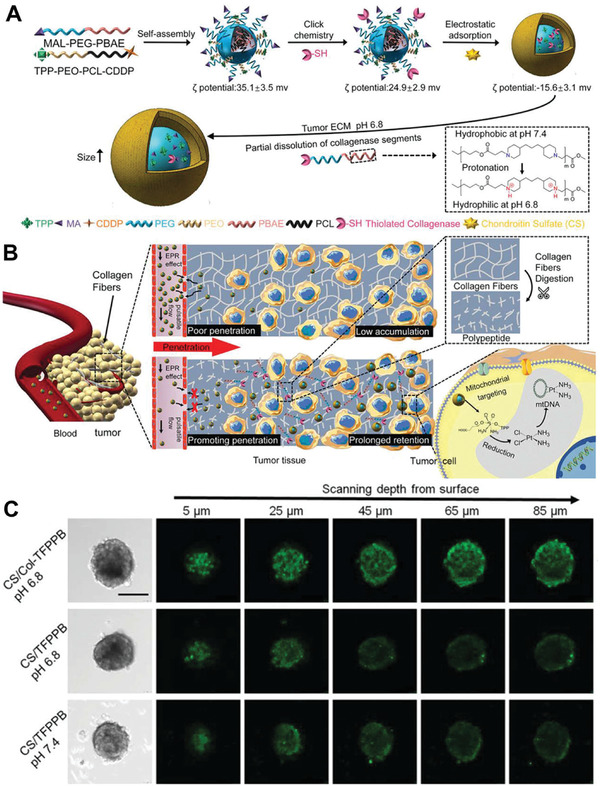
Collagenase modified nanomedicine used to promote deep penetration. A) Preparation process and response mechanism of nanomedicine. B) Penetration promotion effect in solid tumors. C) Penetration effect evaluated in multicellular spheroids. Reproduced with permission.^[^ ^36^ ^]^ Copyright 2020, Wiley‐VCH.

In the surface modification schemes without energy supply, the most common solution is enzyme surface modification, which can dissolve some basic components of the solid tumor microenvironment, such as collagen matrix, thus promoting deep penetration. Collagenase is a surface modification enzyme commonly used for deep penetration.^[^ ^35^ ^]^ Xu et al. modified collagenase on the surface of the nanomedicine under the protection of chondroitin sulfate (**Figure** **3**A).^[^ ^36^ ^]^ The collagenase component was designed to dissociate from the nanomedicine in response to the weak acidity of the tumor microenvironment and produce a deep penetration of up to 85 µm in multicellular spheroids through the digestion of collagen (Figure 3B,C). Hong et al. developed a native PH20 hyaluronidase‐harbored exosome that can deeply penetrate the hyaluronic acid coating of the tumor ECM.^[^ ^37^ ^]^ Applying engineered exosomes to surface modification can maximize the characteristics of enzyme surface functionalization and exosome encapsulation. Such a general engineering strategy can also be applied to the modification of other enzymes and other natural‐state membrane‐bound proteins, with great application prospects. Villegas et al. prepared a nanocarrier with proteolytic enzyme nanocapsule surface modification that can be controllably released to digest the ECM to achieve deep penetration;^[^ ^38^ ^]^ the experimental results showed a great penetration ability in a 200 µm 3D collagen gel model.

The digestibility of surface modification exogenous enzymes can indeed open a channel of deep penetration but its limitations are also obvious. Enzymes are biological macromolecules that can be easily inactivated during the complex and changeable delivery process in vivo. Moreover, direct contact with the delivery environment upon modification on the surface of nanomedicine will increase the risk of inactivation. Therefore, there is a need to protect the surface‐modified exogenous enzymes, for example, through the design of tumor microenvironmental‐responsive nanocapsules to wrap the enzymes. These modifications make the design of nanomaterials more complex and thus do not conform to the purpose of achieving deep penetration through simple and easy surface modification strategies. With this in mind, several studies innovatively degraded the tumor matrix by activating endogenous enzymes.^[^ ^39^ ^]^ Specifically, endogenous MMP‐1 and MMP‐2 were activated by nitric oxide (NO) released by NO donor (*S*‐nitrosothiol) surface modification. With the degradation of the ECM by MMPs and the stability and ease of modification of small molecule NO donors, such surface modification methods have potential to be widely used in the deep penetration of nanomedicine.

The functionalization of nanomedicine surfaces with non‐enzyme molecules, which can also cause damage to the tumor microenvironment, leading to the deep penetration of nanomedicine, has also been performed as a “bulldozer strategy.” Li et al. built a nanosystem with fluorinated chitosan surface modification, which enhanced deep penetration through the conjectural function of transiently opening tight junctions between cells.^[^ ^40^ ^]^ Coincidentally, virus‐derived junction opener protein also possesses the function of opening intercellular junctions transiently in epithelial tumors by cleaving junction protein desmoglein‐2, which can also be used in surface modification for tumor penetration.^[^ ^41^ ^]^

### 3.2 Surface Modification Strategies with Exogenous Energy Supply

Given the complexity and high density of the solid tumor microenvironment, surface modification strategies without requiring exogenous energy may not generate sufficient disruption to the ECM of the solid tumors because of the limited surface area for functionalization. In this case, if the surface modification of the nanomedicine responds to the external energy source and releases the energy at the solid tumor site, more possibilities can be created. Due to the infinite nature of exogenous energy, the nanomaterial can be controlled to release energy continuously until it penetrates deep enough into the solid tumor. To date, the external energy sources for nanomedicine that are widely used include light, ultrasound, and magnetic forces, with considerable progress being made.

#### 3.2.1 Light Energy for Deep Penetration

Photothermal and photodynamic strategies can be used to achieve deep penetration using exogenous light energy as an energy source. In the photothermal strategies, through the photothermal transformation of near‐infrared (NIR) light, a rapid increase in temperature over a short time period can produce very effective damage to solid tumors and promote penetration. Yu et al. grafted a NIR dye, cypate, on a nanosystem to increase the local temperature in response to NIR and produce penetration enhancement while promoting the release of chemotherapeutic drugs.^[^ ^42^ ^]^ Su et al. combined lipid bilayers with sponge‐like graphene nanosheets, causing the rupture of tumor spheroids for penetration promotion.^[^ ^43^ ^]^

Highly toxic reactive oxygen species (ROS) produced due to photodynamic strategies leads to damage to tumor vessels and the ECM. Li et al. modified a photosensitizer, chlorin e6 (Ce6) onto the surface of nanoparticles and showed an obviously deeper penetration under light conditions.^[^ ^44^ ^]^ Yb^3+^ ions also have photodynamic properties comparable to those of traditional photosensitizers and are therefore expected to be modified for solid tumor penetration.^[^ ^45^ ^]^ However, the development of such photothermal and photodynamic strategies remains limited by the poor penetration of NIR.

Studies on deep penetration using photothermal transformation strategies have involved a considerable number of photothermal agents being packaged within nanocarriers for delivery, leading to a reduction in light intensity because of the external enclosure barrier as well as occupying the loading space of other drugs such as chemotherapy and gene drugs, compared with the modification on the surface of nanomedicine. In addition, internal photothermal agents need to be designed for release at the solid tumor site, making the nanosystems complex and impractical.

#### 3.2.2 Magnetic Energy for Deep Penetration

Similar to the process of a bulldozer to clear the roadblock, the mechanical force can often play a key role in the process of deep penetration of nanomedicines.^[^ ^46^ ^]^ A good way to generate mechanical forces is to modify the surface of nanomedicines with magnetic materials and provide energy through exogenous magnetic fields. For example, magnetotactic bacteria are Gram‐negative prokaryotes with an inherent chain of iron oxide nanocrystals that can be used for surface modification; this technique led to 55% of the administered nanomedicine penetrating into hypoxic regions of colorectal xenografts.^[^ ^47^ ^]^ Another magnetism‐mediated deep penetration technique is the use of alternating magnetic fields to increase the temperature of the magnetic material and damage the ECM, with great potential in the field of surface modification.^[^ ^48^ ^]^ However, because the magnetic field weakens rapidly as the distance from the magnet increases, the strategy of deep penetration driven by exogenous magnetic forces has only been used as an auxiliary strategy for superficial tumors and its application scope is greatly reduced due to this limitation. To address this issue, Liu et al. developed a system with two oppositely polarized magnets that achieved a fivefold increase in solid tumor penetration compared to the EPR effect.^[^ ^49^ ^]^

#### 3.2.3 Radiant Energy for Deep Penetration

Radiotherapy is one of the most commonly used tumor therapies in clinical practice.^[^ ^50^ ^]^ Half of all cancer patients receiving radiotherapy alone or in combination, indicating its effectiveness and irreplaceability.^[^ ^51^ ^]^ Given the destructive effects of ionizing radiation on solid tumors and the deeper penetration of rays compared to NIR, the technique can be used to achieve deep penetration of nanomedicine.^[^ ^50a^ ^]^ Furthermore, TAMs have been shown to accumulate in large numbers near the tumor microvasculature after radiotherapy, causing vascular bursts and further promoting tumor site penetration of the nanomedicine.^[^ ^52^ ^]^ Additionally, in order to reduce the damage of high‐intensity radiation to normal tissue, tumor metastasis, recurrence, and resistance to radiotherapy, radiotherapy sensitizers are now widely used.^[^ ^53^ ^]^ In early studies, targeted gold nanoparticles were first delivered as radiotherapy sensitizers to achieve the destruction of vascular endothelium by irradiation.^[^ ^54^ ^]^ However, such a treatment method requires multiple injections or irradiations at certain intervals, which is not easy to achieve compared with integrative nanomedicine containing radiotherapy sensitizer and nanocarrier through surface modification. Although there is a limited number of studies on this, radiotherapy sensitizer surface modification seems promising in opening a channel for the deep delivery of nanomedicine in a manner similar to radiotherapy. However, it is worth noting that radiation therapy often causes damage to tumor blood vessels, which will aggravate hypoxia in the tumor microenvironment and further improve the resistance of hypoxia region cells to chemotherapy and radiotherapy.^[^ ^55^ ^]^

## 4 “Mouse Strategies” of Surface Modification for Nanomedicine Deep Penetration

Mouse, small in size without great strength, can easily move through the soil because of the soft bones that can deform depending on the size and shape of the narrow gaps in the soil. Nanomedicine can also undergo surface modification to produce properties, such as appropriate surface charge and surface softness, which can adapt to the tumor microenvironment and achieve deeper tumor penetration, much like the process of drilling a mouse hole. “Mouse strategies” of surface modification can also be divided into two types, that is, non‐bionic and bionic strategies.

### 4.1 Non‐Bionic Surface Modification Strategies

Non‐bionic strategies change the physical and chemical properties of the surface of nanomedicine, such as surface charge, shape, hydrophobicity, and softness, enabling nanomedicines to adapt to the complex microenvironment of a solid tumor, penetrate into blood vessels and the tumor matrix, and promote tumor cell internalization and deep penetration into solid tumors.

#### 4.1.1 Surface Charge Reversal Strategies

One of the main strategies to enhance the deep penetration and internalization of nanoparticles is surface charge reversal. The positive surface charge of nanomedicines is one of the key factors promoting adsorption‐mediated transcytosis.^[^ ^56^ ^]^ This ATP‐dependent transport mode could help bypass the solid passive‐diffusion barriers and achieve the deep penetration of tumors.^[^ ^57^ ^]^ However, nanomedicines with positive surface charge are easy to be recognized by the reticuloendothelial system in the process of circulation and quickly removed, reducing the efficiency of tumor aggregation. Furthermore, a positive surface charge is toxic to red blood cells and may cause serious coagulation reactions.^[^ ^58^ ^]^ By contrast, neutral or negatively charged nanomedicines are conducive to long blood circulation times but not to the deep penetration of tumor tissue and cellular uptake. Therefore, in order to satisfy both the long‐term circulation times and the optimal tumor permeability, nanomedicines should be able to self‐alter their surface charge with variations in the environment—being either negative or neutral in the circulating blood and becoming positively charged at the tumor site.^[^ ^59^ ^]^ The unique microenvironment of the solid tumor can be used as the trigger condition to achieve charge reversal. Based on the properties of weak acidity, overexpression of enzymes, and hypoxia in the tumor microenvironment, surface charge convertible nanomedicines with corresponding responses have been developed for deep penetration.^[^ ^60^ ^]^

##### pH‐Response Charge Reversal

The pH value in blood and normal tissues is ≈7.4, while the extracellular pH value in solid tumors is ≈6.5.^[^ ^61^ ^]^ This difference in pH value can be used to reverse the surface charge of nanomedicines and the main mechanisms including protonation in an acidic environment and acid unstable chemical bonds break.

During protonation in an acidic environment, many surface charge‐convertible nanomedicines have ionizable groups (such as imidazole and amino groups) that undergo protonation in the weak acid environment of solid tumors and change from negative or neutral to a positive charge. Yang et al. synthesized a pH‐sensitive polymer, poly(ethylene glycol)‐benzoic imine‐poly(*γ*‐benzyl‐l‐aspartate)‐b‐poly(1‐vinyl imidazole) block copolymer (PVIm‐b‐PBLA‐benzoic imine‐mPEG, PPBV) for the co‐delivery of paclitaxel and curcumin.^[^ ^62^ ^]^ In the tumor extracellular environment, at pH ≈ 6.5, the surface charge of the micelle system reversed to positive due to the protonation of imidazole groups of PVIm. Cao et al. designed a nanomedicine consisting of block copolymers of poly(ethylene glycol) and poly(trimethylene carbonate) (PEG–PTMC) that converted to a positive charge due to protonation at the low pH of the solid tumor, with the potential to promote tumor penetration.^[^ ^63^ ^]^ Furthermore, poly(2‐ethyl‐2‐oxazoline) (PEOz), as a substitute for PEG, can produce a significant pH‐responsive charge reversal effect after surface modification, with promising application prospects for deep tumor penetration.^[^ ^64^ ^]^

**Figure 4:**
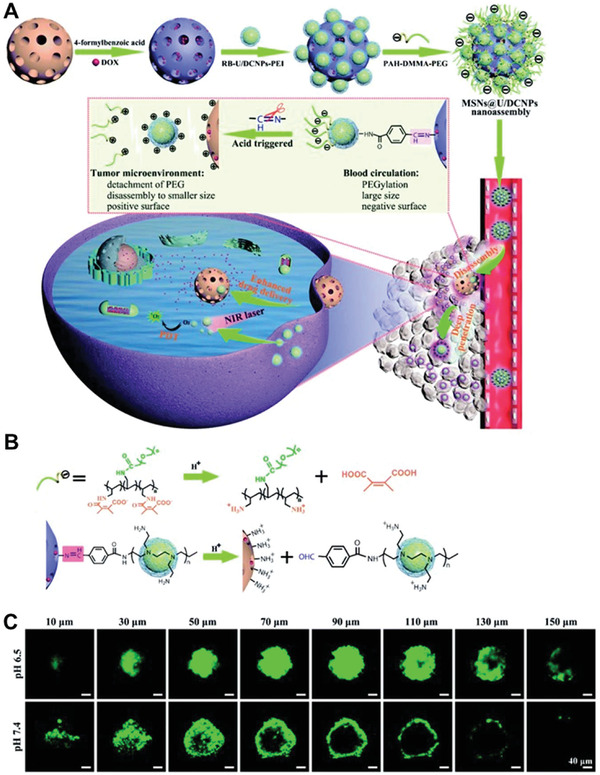
A) Schematic illustration of the preparation process of core@satellite nanoassemblies. B) Mechanism of multiple charge reversal. C) Tumor penetration of MSN@U/DCNPs in multicellular spheroids at pH 6.5 and 7.4. Reproduced with permission.^[^ ^65^ ^]^ Copyright 2020, Royal Society of Chemistry.

Other pH sensitive strategies include acid unstable chemical bond breakage of chemical bonds such as amide (2,3‐dimethylmaleic anhydride, succinic anhydride) and imine (benzoic imine) bonds that can break at low pH microenvironment for a penetration‐promoting effect of charge reversal. Chen et al. reported a nanoassembly of core@satellite structure of mesoporous silica nanoparticles (MSN) and up/down converting nanoparticles (U/DCNPs).^[^ ^65^ ^]^ Small size U/DCNPs were connected with MSN via acid unstable benzoic‐imine bonds. In addition, a pH‐responsive charge reversible polymer layer, poly(allylamine)–dimethylmaleic anhydride–polyethylene glycol (PAH–DMMA–PEG), was modified on U/DCNPs. At the acidic tumor microenvironment, PAH–DMMA–PEG and the benzoic‐imine bonds between MSN and U/DCNPs were broken, causing a surface charge reversal of MSN and U/DCNPs from negative to positive (**Figure** **4**A,B). The experimental results showed that the positively charged surface improved the tumor penetration and internalization efficiency of MSNs and U/DCNPs (Figure 4C). Dai et al. used the pH‐sensitive amide bond (pKa 6.8) of 2‐propionic‐3‐methylmaleic anhydride (CDM) for charge reversal.^[^ ^66^ ^]^ At the weak acid environment, the amide bonds were cleaved and a positive PEI layer was exposed. The real‐time 3D distribution images of tumor tissue showed that the designed nanomedicine was evenly distributed throughout the tumor, even if the depth of the tumor was 300 µm, whereas the nanomedicine in the control group was only distributed in the surrounding tumor tissue.

pH‐responsive charge reversal surface‐modified nanomedicines have the advantage of simple preparation and the obvious effect of promoting cell internalization. However, due to the heterogeneity of the solid tumor microenvironment, there are differences in the pH value of solid tumors in different patients. Similarly, the acidity of the tumor microenvironment is weak, and not much different from the normal physiological environment, requiring an extremely high sensitivity to pH changes to induce charge reversal. In addition, the vessels are still some distance from the tumor microenvironment, leading to a decrease in the effectiveness of a pH‐responsive charge reversal strategy. In general, surface modification strategies based on pH‐responsive charge reversal still have some room for improvement.

##### Enzyme‐Response Charge Reversal

**Figure 5:**
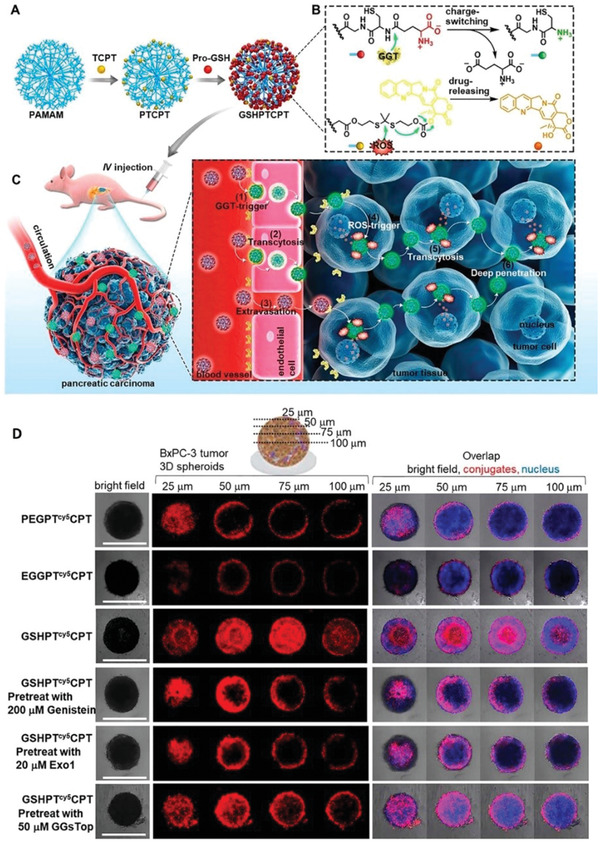
A) Schematic illustration of the preparation of nanomedicine. B) GGT‐catalyzed charge reversal process. C) The positive charge conjugates internalized rapidly through vesicle‐mediated endocytosis and enhanced deep penetration into the tumor parenchyma. D) Comparison of penetration effect. Reproduced with permission.^[^ ^67^ ^]^ Copyright 2020, American Chemical Society.

The over‐expressed enzymes in the tumor microenvironment can be used as stimuli to achieve surface charge reversal and can be more specific compared to pH. Based on the high expression of *γ*‐glutamyl transpeptidase (GGT) in endothelial and tumor cells near the blood vessels, Zhou et al. designed a GGT‐responsive polymer nanomedicine for the delivery of camptothecin (CPT).^[^ ^57a^ ^]^ The polymer showed a neutral charge in blood; yet, upon contact with the endothelial or tumor cells, the over‐expressed GGT hydrolyzed the *γ*‐glutamyl group to produce an amino group, rendering the polymer positively charged, and promoted the vascular epidermal or tumor cells to rapidly internalize the cationic compounds, promoting endothelialization of the nanomedicine and deep delivery to tumor cells. Similarly, Wang et al. covalently linked CPT to poly‐amidoamine via ROS‐sensitive linkers and then modified them with glutathione (GSH) to synthesize dendrimer drug conjugates (GSHPTCPT) (**Figure** **5**A).^[^ ^67^ ^]^ In the blood circulation, the amphoteric glutamate residues on the surface had a negative charge, yet upon reaching the periphery of pancreatic ductal adenocarcinoma tumor tissue, overexpressed GGT‐mediated charge reversal enhanced the deep penetration into the tumor parenchyma (Figure 5B,C). Cy5‐labeled conjugates were incubated with BxPC‐3 MTS for 6 h to evaluate the in vitro tumor penetration. Compared with the control groups, GSHPTCy5CPT penetrated more deeply into the spheroids (Figure 5D).

Among the surface modification charge reversal strategies, the GGT response is the most effective by far. The high expression of GGT not only on the solid tumor cells but also on nearby blood vessels endothelial cells. Such a distribution characteristic enables it to achieve charge reversal sensitively, which is essential for deep penetration. In conclusion, this is a promising surface modification strategy for tumor treatment.

##### Hypoxia‐Response Charge Reversal

**Figure 6:**
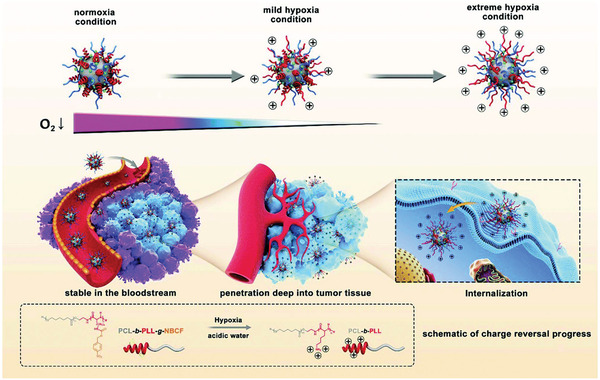
Schematic diagram of the hypoxia‐responsive charge reversal nanomedicine. Reproduced with permission.^[^ ^68^ ^]^ Copyright 2019, Royal Society of Chemistry.

The tumor microenvironment is characterized by hypoxia and the concentration of oxygen decreases significantly with the deepening of the tumor. Therefore, the tumor oxygen gradient can be used as a driving force to achieve the deep penetration of nanomedicines. Zhen et al. developed a hypoxia‐responsive nanomedicine composed of a poly(caprolactone) core and a PEG and 4‐nitrobenzyl chloroformate (NBCF)‐modified polylysine (PLL)‐mixed shell that can increasingly reverse to positive surface charge through a response to the hypoxia gradient, finally reaching the inner tumor (**Figure** **6**).^[^ ^68^ ^]^ After arriving at the tumor tissue, the hypoxia microenvironment led to the degradation of part of the NBCF, exposing the amino group of PLL, leading to a surface charge reversal to positive. With the further decrease in central oxygen concentration in solid tumors, NBCF‐modified PLL was further degraded and the surface positive charge increased, further promoting the deep penetration of tumors.

In addition to pH, enzymes, and hypoxia‐response charge reversal, other tumor microenvironment characteristics or exogenous condition‐mediated charge reversals, such as ROS,^[^ ^69^ ^]^ GSH,^[^ ^70^ ^]^ hydrogen sulfide,^[^ ^71^ ^]^ and thermal,^[^ ^72^ ^]^ have been investigated but their effects in achieving deep penetration of nanomedicines have not yet been verified.

In general, charge reversal‐mediated active transport strategy utilize the process of active endocytosis and transport of tumor cells, which can allow the nanomedicine to penetrate into the whole tumor and reach the distal cells without the restriction of the passive diffusion barrier. By avoiding the natural biological barrier formed by the dense microenvironment of tumor tissue, active penetration can overcome the natural defect of the low diffusion ability caused by the large size of nanomedicines, thus breaking new ground for the design of drug delivery systems.

#### 4.1.2 Surface Deformation Strategies

Due to the complex tumor composition and abnormal environment mentioned above, the gaps between tumor cells and the dense ECM are very narrow, such that most of the nanomedicine cannot pass through due to a shape mismatch and thus cannot achieve deep penetration. Previous studies have shown that nanomedicines of different shapes have varying penetrability.^[^ ^73^ ^]^ Similarly, considering the different environment and types of resistance encountered by nanomedicines in the blood circulation and tumor penetration, the nanomedicine that can change its shape at different stages of delivery is more likely to meet the therapeutic needs. Similar to how mice get into deep caves, nanomedicines with shape‐shifting properties are more likely to penetrate deep within tumors.

##### Mechanical Pressure Response Surface Deformation

**Figure 7:**
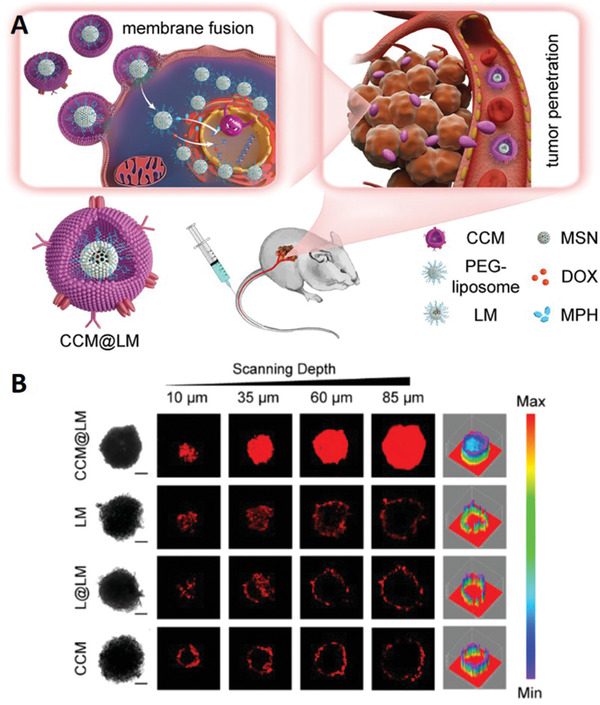
A) Schematic illustration of CCM@LM. B) Penetration of nanoparticles in MCF‐7 multicellular spheroids (MCSs). Reproduced with permission.^[^ ^75^ ^]^ Copyright 2020, American Chemical Society.

The properties of surface deformation in response to mechanical pressure can be attributed to surface elasticity, which has become an important parameter in nanocarrier design. The flexibility of nanoparticles can affect their blood circulation, tumor penetration, and endocytosis.^[^ ^74^ ^]^ Because of the high heterogeneity of tumor blood vessels and stroma, elastic nanomedicines have a stronger ability to pass through blood vessels and penetrate into the tumor parenchyma than rigid nanomedicines because of their deformability. A significant degree of elasticity of nanomedicines can be achieved by coating a layer of materials with certain elasticity, such as cell membranes, vesicles, or liposomes. Nie et al. designed a yolk‐shell nanoparticle with an MSN‐supported PEGylated liposome core and cancer cell membrane (CCM) coating (CCM@LM) (**Figure** **7**A).^[^ ^75^ ^]^ The yolk–shell structure endowed CCM@LM with moderate rigidity and elasticity, which might help to transform into an ellipsoidal shape frequently during tumor penetration, which is embodied obviously in the multicellular spheroid model (Figure 7B). In another study,^[^ ^76^ ^]^ extracellular microparticles (MPs) with a softness derived from tumor‐repopulating cells (TRCs) were used as drug carriers to achieve the deep penetration of tumors. TRCs were prepared in soft 3D fibrin gels of 90 Pa in stiffness, so that MPs derived from these TRCs were soft and deformable enough for exosmosis, penetration into tumor tissues, and internalization into TRCs. The results showed a significant penetration in the soft 3D fibrin gel tumor spheroid model in vitro. Wu et al. adjusted the rigidity of the liposome membrane by changing the cholesterol content of liposomes to achieve a moderate hardness for penetration.^[^ ^77^ ^]^ The results showed that, when the molar ratio of hydrogenated soybean phospholipids (HSPC) to cholesterol to 1,2‐distearoyl‐sn‐glycero‐3‐phosphoethanolamine‐*N*‐[methoxy(polyethylene glycol)‐2000] (DSPE‐PEG_2000_) was 67.3:30.1:2.6, the liposome membrane had a moderate hardness and good penetration in the 3D tumor spheroids.

Regulating the surface elasticity of nanomedicines can increase their passive diffusion, allowing them to achieve greater penetration depth in a solid tumor environment. However, extremes in elasticity are not conducive to the deep penetration into the tumor, thus a suitable material ratio of different surface‐modified substances must be achieved for moderate elasticity, which is time consuming.

##### Other Responses to Surface Deformation

In addition to the deformation caused by the pressure of the tumor matrix and cells, the shape of a nanomedicine can also be changed through a response to other conditions. Chen et al. synthesized a class of nanoaggregates based on BF2‐azadipyrromethene (aza‐BODIPY) dyes that could deform between wormlike nanofibrous and spherical nanoparticles under a NIR laser.^[^ ^78^ ^]^ Wormlike nanofibrous aggregates were beneficial to achieve long blood circulation times, while spherical nanoparticles were beneficial for tumor penetration. An et al. presented a tumor‐selective cascade activatable self‐detained system consisting of a tumor‐specific recognition motif, an enzymatically cleavable linker, a self‐assembly motif, and a functional molecule (cyanine dye or doxorubicin).^[^ ^79^ ^]^ X‐linked inhibitor of apoptosis protein, which was highly expressed in tumor cells, was recognized by the designed system. Then, caspase‐3/‐7 was activated in the recognition process, cleaving the designed molecules, which then rapidly self‐assembled in situ. Hydrogen bonds guided the growth of the assembly and, finally, *β*‐sheet nanostructures were formed. The *β*‐sheet nanostructures obviously improved the accumulation and retention properties of functional molecules in tumor tissues.

#### 4.1.3 Size Change‐Related Surface Modification Strategies

Nanomedicines with a relatively large size (100–200 nm) have prolonged blood circulation times but fail to penetrate within the deep tumor parenchyma; in contrast, smaller nanoparticles (<30 nm) can easily penetrate the tumor but are usually rapidly cleared.^[^ ^80^ ^]^ Therefore, a nano delivery system with variable size is required, wherein a large initial particle size is maintained in the blood to achieve long circulation times and then degrades to a smaller particle to promote deep penetration. The strategies to achieve this goal through surface modification are divided into two main types: surface‐responsive transformation‐mediated size changes and the release of smaller surface‐modified particles.

##### Surface Transformation‐Mediated Size Change

**Figure 8:**
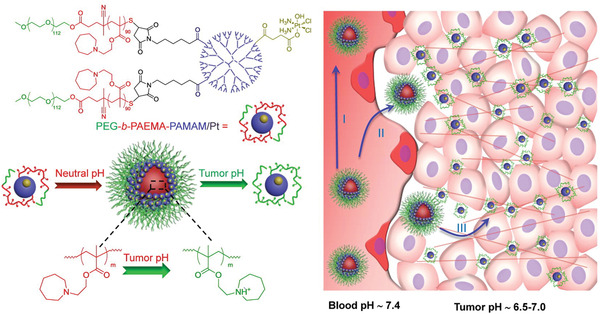
Structure of PEG‐b‐PAEMA‐PAMAM/Pt and the mechanism of deep penetration. Reproduced with permission.^[^ ^81^ ^]^ Copyright 2016, American Chemical Society.

The modified surface layer can be removed or shrunk under specific conditions, promoting an intelligent size change of a nanomedicine, with a potentially great promoting effect on solid tumor penetration. Li et al.^[^ ^81^ ^]^ developed a pH‐responsive shrinkable nanoparticle system self‐assembled by poly(ethylene glycol)‐*b*‐poly(2‐azepane ethyl methacrylate)‐modified poly‐amidoamine dendrimers (PEG‐*b*‐PAEMA‐PAMAM). At the neutral pH of blood circulation, PAEMA was hydrophobic and the superstructures had a size of 80 nm. In the tumor environment at pH ≈6.5, PAEMA particles rapidly protonated and became hydrophilic, reducing in size to less than 10 nm, suitable for deep tumor penetration (**Figure** **8**). Tong et al. developed spiropyran‐based nanoparticles, which can produce sharp shrinkages under UV radiation of 365 nm.^[^ ^82^ ^]^ UV light induced the conversion of hydrophobic spiropyran to amphoteric merocyanine, which changed the physical assembly characteristics of nanoparticles and reduced their volume, enhancing their tumor penetration.

In order to reduce the size of nanoparticles, their surface shell layer can be separated by responding to the tumor microenvironment, thus forming smaller nanoparticles. In the study of Chen et al., a kind of pH‐sensitive shell‐stacked nanoparticle (SNP) was reported.^[^ ^83^ ^]^ The DMMA‐modified polypeptide was used as a detachable shell. Due to the effect of electrostatic shelling, the size of the nanoparticles in acid tumor tissue was considerably reduced, from 145 to 40 nm, and the surface charge was changed from −7.4 to 8.2 mV, thus enhancing the penetration and absorption of tumor cells. Hu et al. prepared hyaluronidase (HAase)‐sensitive, size‐changeable nanoparticles composed of a hyaluronic acid (HA) shell and a conjugated dendrimer core.^[^ ^84^ ^]^ The high expression of HAase in the tumor microenvironment led to the degradation of the HA shell; after 4 h of incubation with HAase, the nanoparticles rapidly degraded from 330 nm to 35–150 nm.

##### Release of Smaller Surface‐Modified Particles

**Figure 9:**
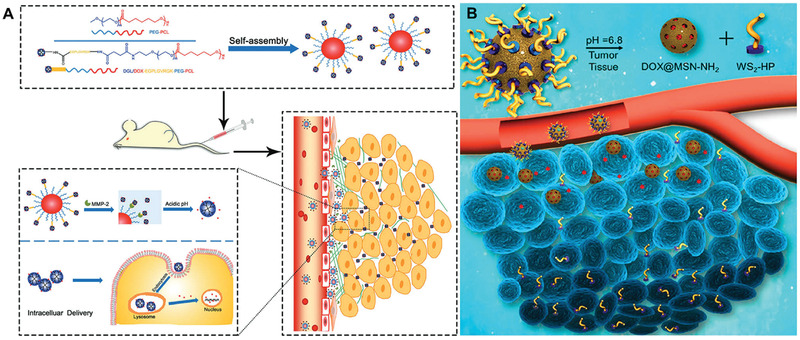
Release of smaller surface‐modified particles for deep penetration. A) Schematic diagram of DGL/DOX@PP and DGL release for deep penetration. Reproduced with permission.^[^ ^85^ ^]^ Copyright 2018, Royal Society of Chemistry. B) Schematic diagram of WS_2_‐HP release for deep penetration. Reproduced with permission.^[^ ^86^ ^]^ Copyright 2017, American Chemical Society.

Carrying smaller nanoparticles on the surface of nanomedicine through a tumor microenvironment sensitive bond is a kind of common size change strategy. In tumor tissue, with the breakage of tumor microenvironment sensitive bond, the connected small nanoparticles are rapidly released. Like a bomber dropping bombs, such a strategy allows the deep penetration of nanomedicines into deep tumors. Cun et al. prepared a size‐switchable nanoplatform (DGL/DOX@PP) by combining small dendrigraft poly‐L‐lysine (DGL) with poly(ethylene glycol)–poly(caprolactone) micelles via a MMP‐2‐sensitive peptide.^[^ ^85^ ^]^ After extravasation, the peptides in DGL/DOX@PP were cut by MMP‐2 in the tumor microenvironment and small DGL/DOX nanoparticles were rapidly released, showed an enhanced penetration in multicellular spheroids and solid tumors (**Figure** **9**A). Lei et al. developed small size tumor‐homing/‐penetrating peptide tLyP‐1‐modified tungsten disulfide quantum dots (WS_2_‐HP) with a good photothermal conversion efficiency and the ability for deep tumor penetration. These quantum dots were connected to DOX‐loaded mesoporous silica nanoparticles via acid‐labile benzoic–imine bonds.^[^ ^86^ ^]^ At pH 6.8, the benzoic–imine bonds broke and smaller WS_2_‐HP were released to achieve deep tumor penetration (Figure 9B). In another study,^[^ ^87^ ^]^ gold nanorods were loaded on the surface of polydopamine nanospheres. Under NIR irradiation, the nanomedicine was decomposed and the small‐scale gold nanorods dropped from the original nanocarrier and entered the internal tissue of the tumor, achieving deep penetration.

Compared with nanomedicines with a surface transformation‐mediated size change, nanomedicines with small nanoparticles on the surface may carry more functional materials, yet there are issues with their complex design.

### 4.2 Bionic Surface Modification Strategies

In nature, bacteria, viruses, or certain functional cells have the ability to penetrate tumors in depth due to their inherent surface properties. Thus, biomimetics is a clever and practical surface modification strategy to achieve the deep penetration of solid tumors. Surface bionics can either mimic only some substances on the surface of living entities to promote penetration (partial bionic strategy) or apply the whole outer layer of the living entities (such as cell membrane and virus shell) for nanomedicine surface modification (complete bionic strategy).

#### 4.2.1 Partial Bionic Surface Modification Strategies

Partial bionic surface modification strategies imitate the endogenous substances and processes utilizing over‐expressive receptors or mimicking essential nutrients, such as albumin, to enhance the tropism of deep tumors.

##### Mimicking the Nutrients Tumors Need and Using Overactivated Transport Processes

Albumin is one of the key nutrients for tumor growth. In order to obtain enough amino acids and energy, albumin transporters are over‐activated to preferentially absorb albumin in the blood, which promotes the growth of tumor cells.^[^ ^88^ ^]^ Therefore, the use of albumin and its transport process can improve the absorption and penetration of nanomedicine. Based on the high expression of albumin‐binding proteins (SPARC and gp60) in glioma cells and tumor neovascular endothelial cells, Lin et al. synthesized albumin nanoparticles for paclitaxel and fenretinide loading.^[^ ^89^ ^]^ Albumin nanoparticles can target glioma cells through biomimetic transport mechanism mediated by SPARC and gp60, with the nanoparticles showing a wide range of penetration in the spheroids. Due to the natural abundance of albumin in the blood, nanomedicines with albumin surface modification may have lower immunogenicity and can be preferentially absorbed by the tumor cells. However, because normal cells also express albumin‐binding protein, this strategy may also damage normal cells. Further improvement of tumor specificity is an important development direction for the albumin surface modification strategy.

Ferritin is the main iron transport and storage protein in prokaryotes and eukaryotes. Transferrin receptor 1 is up‐regulated in most cancers and transports ferritin; therefore, this over‐activated transport process can be used to achieve deep tumor penetration.^[^ ^90^ ^]^ Huang et al. designed PEGylated human ferritin heavy chain nanocages (FTn) in which DOX was bound to the PEGylated FTn via an acid‐sensitive linker.^[^ ^90^ ^]^ PEGylated FTn preferentially penetrated and was distributed in lung cancer tissues in situ in a transferrin receptor‐1‐dependent manner and selectively entered cancer cells. The confocal images of 3D reconstruction showed that the free DOX was mainly around the spheroids, while FTn/FTn–PEG2k/DOX was evenly distributed throughout the tumor spheroids. In another study, transferrin was used to enhance the tumor penetration of polysulfamide‐based (poly(2‐((2‐(methacryloyloxy)ethyl) dimethylammonio)acetyl) (phenylsulfonyl) amide) nanogels.^[^ ^91^ ^]^ These nanogels showed a favorable penetration ability in 3D tumor spheroids.

##### Using Tumor‐Homing Peptides and Penetrating Peptides

Tumor‐homing peptides (such as iNGR, iRGD, and Lyp‐1) and tumor‐penetrating peptides (such as cell‐penetrating peptide (CPP)) have been reported to have tumor homing and tumor penetrating capabilities.^[^ ^25^, ^92^ ^]^ In recent years, the research of tumor‐homing and tumor‐penetrating peptides has mainly focused on tumor targeting and tumor penetration by using their tumor‐homing ability and on the modification of peptides to obtain a better tumor microenvironment response.

**Figure 10:**
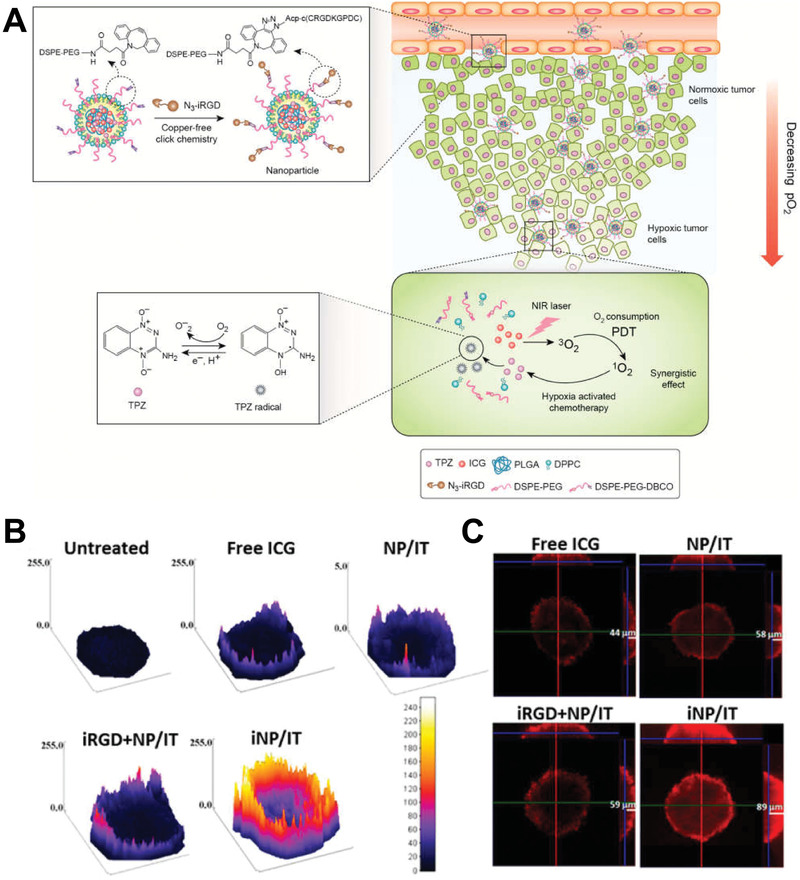
A) Schematic representation of iRGD modified nanoparticles. B) Surface plot images of 4T1 spheroids treated with free ICG, NP/IT, iRGD+NP/IT, iNP/IT, and negative control. C) Confocal microscopy images of 4T1 spheroids in *X*, *Y*, and *Z* direction after different treatments. Reproduced with permission.^[^ ^95^ ^]^ Copyright 2017, American Chemical Society.

iRGD, a disulfide bridged cyclic peptide, first recognizes the tumor site by binding to the up‐regulated *α*v*β*3/*α*v*β*5 integrin in vascular endothelial cells or tumor cells.^[^ ^93^ ^]^ After cleavage by a proteolytic enzyme, the peptide binds to the second receptor neuropilin‐1 or neuropilin‐2 to activate the transport pathway. This cross‐tissue pathway is called C‐end rule (CendR), and can mediate the transport of nanomedicine through extravascular tumor tissue.^[^ ^94^ ^]^ The amino acid sequence alanine‐alanine‐asparagine was covalently bound to the tumor‐homing peptide iRGD (CCRGDKGPDC) to obtain nRGD, and used for the surface modification of DOX‐loaded liposomes to penetrate deep into the tumor tissue.^[^ ^92^ ^a]^ Wang et al. developed iRGD‐modified nanoparticles for the delivery of the photosensitizer indocyanine green (ICG) and of the hypoxia‐activated prodrug tirapazamine to treat breast cancer.^[^ ^95^ ^]^ The penetration of iRGD‐modified nanoparticles was significantly improved (**Figure** **10**). In another study, Wang et al. modified nanoparticles made of natural high‐density lipoproteins (HDLs) with the tumor‐penetrating peptide iRGD and loaded them with paclitaxel (PTX) and ICG (pHDL/PTX‐ICG) for synergetic chemo‐phototherapy.^[^ ^96^ ^]^ After intravenous injection of pHDL/PTX‐ICG, iRGD mediated the binding with *α*v integrins on tumor endothelial cells and was then hydrolyzed and cleaved, exposing the binding site of Nrp‐1, and finally promoting tumor penetration. Compared with the control group, iRGD‐modified nanoparticles had better tumor penetration in A549 tumor spheroids.

The iRGD peptide can promote the extravasation and transport of nanomedicine in tumor tissue through the transorganizational pathway mediated by CendR, which provides a solution for enhancing the penetration of nanomedicine in solid tumors.

CPP can target intracellular proteins, and can be divided into three types: cationic, amphipathic, and hydrophobic.^[^ ^97^ ^]^ CPP functionalized with GBI‐10 aptamer, which has a high affinity to the ECM component tenascin‐C, was linked to the surface of nanoparticles.^[^ ^92^ ^b]^ Tenascin‐C separated the GBI‐10 aptamer from CPP and the exposed CPP promoted further endocytosis of tumor cells. Apt/CPP‐CPTD‐NPs showed deep penetration in 3D spheroids in vitro and in tumor sections in vivo. Ding et al. grafted CPPs under an acid response layer to avoid the interaction with normal tissues.^[^ ^98^ ^]^ The acid response layer was composed of poly(*β*‐aminoester) and PEG. The acidic tumor microenvironment can trigger the exposure of CPPs, promoting deep tumor penetration. Additionally, trans‐activating transcriptional activator (TAT) peptide, a type of CPP, has been widely used to deliver a variety of nanomedicines across cell membranes and can be used for deep tumor penetration. Liu et al. used a one‐pot synthesis method to conjugate the cytotoxic peptide KLAK, TAT, matrix MMP‐2‐sensitive peptide, and PEG onto dendrimers to obtain PKT‐S‐PEG;^[^ ^99^ ^]^ the multicellular spheroid showed good tumor penetration.

Although CPPs can improve the penetrating ability of nanomedicines, CPPs need to be modified. For example, for cationic CPPs, acid‐responsive reversible PEGylation can be used to improve their blood circulation time but increasing the complexity of nanomedicine. In addition, CPP undergoes non‐specific interactions, which requires ligand modification to increase the specific response to the tumor microenvironment.

#### 4.2.2 Complete Bionic Surface Modification Strategies

Complete bionic surface modification strategies modify nanomedicines by using the biological structure of living entities with tissue permeability to achieve the deep penetration of tumors. The complete bionic strategy can be achieved by using cells or cell derivatives with permeability or by imitating the structure of viruses or bacteria to modify the nanomedicine surface.

##### Bionic Strategies Based on Cells

**Figure 11:**
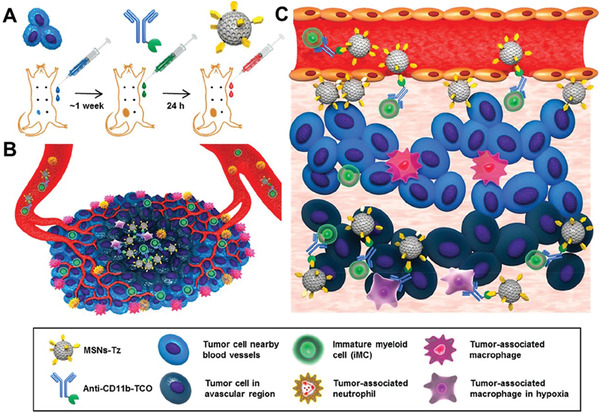
A) The treatment process of nanomedicine. B,C) Schematic illustration of CD11b+ cells modified MSNs‐TZ for deep tumor penetration. Reproduced with permission.^[^ ^100^ ^]^ Copyright 2019, American Chemical Society.

T cells, macrophages, neutrophils, and other immune cells can actively migrate to the target tissue, cross the biological barrier, and penetrate into the infected site.^[^ ^25^ ^]^ Because tumors often occur in chronic inflammatory sites, immune cells will preferentially be recruited into the tumor microenvironment.^[^ ^100^ ^]^ Lee et al. reported a strategy to penetrate tumors assisted by immunocytes. Inflammatory CD11b+ cells were used as active carriers to deliver DOX‐loaded nanoparticles to areas with poor tumor vascularization.^[^ ^100^ ^]^ Trans‐cyclooctene‐modified CD11b antibodies were used as a connector to bind CD11b^+^ cells to the surface of 1,2,4,5‐tetrazine (TZ)‐functionalized mesoporous silica nanoparticles (MSNs‐TZ) in vivo (**Figure** **11**). In the avascular region of the tumors, the accumulation of CD11b+ cells functionalized MSNs‐TZ transferred by immunocytes was twofold higher than that of nanoparticles transferred by the EPR effect.

Bone marrow‐derived monocytes/macrophages can also be used as carriers to improve the distribution of nanoparticles in tumors, especially in the hypoxic area. Huang et al. used bone marrow‐derived monocytes/macrophages of C57BL/6J mice as cell carriers to co‐deliver echogenic polymer/C5F12 bubbles and DOX‐loaded polymers in a tumor hypoxia environment.^[^ ^101^ ^]^ Such a cell‐mediated nanocarrier can easily penetrate from the nearest blood vessel into the tumor to a depth beyond 150 µm, while the depth of nanoparticles in the control group was limited to ≈10–15 µm.

Bacteria have the ability to navigate autonomously according to chemical gradients and can penetrate dense tissues. Anaerobic bacteria, in particular, can colonize the anoxic area of tumor tissue and reach the internal area of malignant tumors. Moreno et al. used *Escherichia coli* to modify nanoparticles to achieve deep tumor penetration.^[^ ^102^ ^]^ *E*. *coli* was modified on the surface of DOX‐loaded MSNs by a click reaction. In a 3D tumor matrix model composed of collagen ECM containing human fibrosarcoma cells, bacteria‐modified nanoparticles destroyed nearly 80% of the tumor cells under the thick collagen layer.

MPs are a kind of phospholipid bilayer membrane structure with a diameter of 100–1000 nm, produced by budding on the cell membranes in response to internal/external stimulation.^[^ ^103^ ^]^ MPs have unique advantages, such as low immunogenicity, homing and targeting abilities, and breaking through the physiological barrier of tumor treatment, and can therefore be used in drug delivery. Wang et al. used cell‐derived MPs to modify nanoparticles to achieve deep tumor penetration.^[^ ^103^ ^]^ The MPs were prepared by co‐embedding Bi_2_Se_3_ nanodots and DOX into tumor cells and inducing tumor cells to sprout by UV irradiation. Bi_2_Se_3_/DOX@MPs increased cell internalization and deepened tumor invasion through membrane fusion. The 3D tumor spheroid experimental results showed that the DOX fluorescence intensity of Bi_2_Se_3_/DOX@MP‐treated tumor spheroids at a depth of 15 and 35 µm was 6.7‐times and 9.1‐times higher than that of the free DOX‐treated spheroids, respectively.

Exosomes can also be used for the surface modification of nanomaterials to achieve deep tumor penetration. Pan et al. developed PMA/Au‐BSA@Ce6 nanoparticles loaded into exosomes (Exo‐PMA/Au‐BSA@Ce6) extracted from urine by instantaneous electroporation.^[^ ^104^ ^]^ Ce6 fluorescence was observed in every tumor section of the Exo‐PMA/Au‐BSA@Ce6‐treated group even at a depth of 90 µm, indicating that Exo‐PMA/Au‐BSA@Ce6 had a strong penetration ability in vitro. Yong et al. prepared bionic porous silicon nanoparticles (PSiNPs) based on tumor exosomes.^[^ ^105^ ^]^ The exosome‐sheathed DOX‐loaded PSiNPs (DOX@E‐PSiNPs) were produced by tumor cells that first swallowed DOX‐loaded PSiNPs and then proceeded with exocytosis. Confocal fluorescence microscopic images clearly showed that DOX@E‐PSiNPs were widely distributed in the whole tumor tissue 24 h after injection. Similarly, Zhu et al. used electroporation to prepare tumor‐exocytosed exosome/aggregation‐induced emission luminogen hybrid nanovesicles to achieve tumor penetration and photodynamic therapy.^[^ ^106^ ^]^

Among cell‐based biomimetic strategies, the use of living cells such as *E*. *coli* can lead to good tumor microenvironment localization and penetration but cannot guarantee the activity of cells after modification, which can lead to cell death and uneven activity. In addition, heterologous living cells may activate immunity. In contrast, it is safer and easier to modify nanomedicine with cell membranes or vesicles secreted by cells.

##### Bionic Strategies Based on Virus Structure

Virus particles naturally invade host cells. The structure of viruses enables them to achieve effective tissue penetration, rapid attachment to cell membranes, and endocytosis into host cells. For viruses, an acidic environment of pH ≈ 6.5 is an important trigger factor for virus penetration, which is fully matched with the acidic tumor microenvironment.^[^ ^107^ ^]^ Viruses such as rabies and tobacco mosaic virus (TMV), which have a unique rod‐shaped morphology, are used for drug delivery, gene transfer, and tumor imaging, because their high aspect ratio morphology is conducive to long cycles, tumor targeting, tumor penetration, and cellular uptake.^[^ ^108^ ^]^

**Figure 12:**
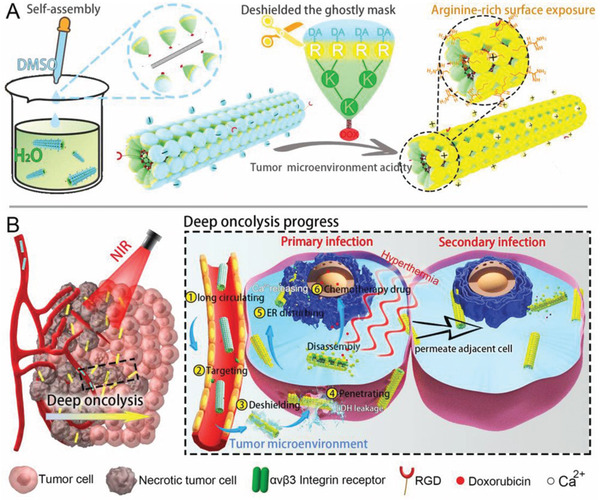
A) Assembly of artificial tobacco mosaic virus nanoparticles (ATMVs). B) Process of deep penetration of ATMVs. Reproduced with permission.^[^ ^108^ ^]^ Copyright 2020, Wiley‐VCH.

An artificial TMV (ATMVs) nanoparticle with a very similar structure to the rod‐shaped TMV can specifically infect and lyse malignant tumor cells and achieve deep tumor penetration (**Figure** **12**).^[^ ^108^ ^]^ ATMVs were prepared by repeated subunits of capsid‐mimicking dendrons self‐assembling onto RGD‐modified, single‐walled carbon nanotubes. The ATMVs not only lysed the primary infected cells but also infiltrated the adjacent cells for secondary infection, which spread from cell to cell and even continued to induce lysis in the deep solid tumors. Zhang et al. synthesized dendrimer nanoparticles that imitated the membrane‐breaking structure of the virus. A special peptide precursor nanomedicine, dendritic and rich in arginine, was designed to mimic the viral protein transduction domain and globular protein structure.^[^ ^109^ ^]^ Tumor‐specific acidic conditions activated the membrane‐breaking ability of these virion‐like nanomedicines, thus penetrating artificial and natural membrane systems. In the tumor microenvironment, virion‐like nanomedicines undergo cell and tissue penetration and can infiltrate into other tumor cells from endothelial cells and tumor cells to achieve vascular extravasation and deep tumor invasion. Experimental results showed that virion‐like nanomedicines can effectively penetrate into drug‐resistant human ovarian cancer cells.

Compared with other methods to promote tumor penetration, the strategies of imitating viral structure may have unique advantages in simulating the process of virus infection and spreading among tumor cells. More detailed information on complete bionic surface modifications is shown in **Table** **1**.

**Table 1:** Complete bionic nano system for deep penetration

Surface modifier	Nano system	Penetration evaluation model in vitro	Penetration evaluation animal model	Reference
Inflammatory CD11b+ cells	Anti‐CD11‐TCO/MSNs‐Tz		4T1 tumor mouse model	^[^ ^100^ ^]^
Bone marrow derived monocytes	PB/DLPV@monocyte		Tramp‐C1 mouse model	^[^ ^101^ ^]^
Inflammatory monocytes	M‐SMNs		4T1 lung metastatic breast cancer model	^[^ ^110^ ^]^
*Escherichia coli* bacteria	Bac‐MSN	3D Collagen Tumoral Matrix Model		^[^ ^102^ ^]^
Cell‐derived microparticles (MPs)	Bi_2_Se_3_/DOX@MPs	Multicellular tumor spheroids (H22 cells)		^[^ ^103^ ^]^
Exosomes	Exo‐PMA/Au‐BSA@Ce6	3D Multicellular tumor spheroids (MCTS) of MGC‐803	MGC‐803 mouse model	^[^ ^104^ ^]^
Exosomes	DOX@E‐PSiNPs	H22 tumor spheroids	H22 tumor mouse model	^[^ ^105^ ^]^
Exosomes	EXO/AIEgen nano‐vesicles		4T1 tumor mouse model	^[^ ^106^ ^]^
Cancer cell membrane	CCM@LM‐DOX‐MPH	Multicellular tumor spheroids (MCF‐7 cells)	MCF‐7 xenograft tumor model	^[^ ^75^ ^]^
Extracellular microparticles	DOX@3D‐MPs	Multicellular tumor spheroids (H22 cells)	H22 tumor mouse model/B16‐F10 tumor zebrafish embryo model	^[^ ^76^ ^]^
Liposomes		BxPC‐3 and HPaSteC co‐cultured 3D tumor spheroids	BxPC‐3 and HPaSteC tumor nude mouse model	^[^ ^77^ ^]^
Capsid‐subunit‐mimetic dendrons (CSMDs)	Artificial tobacco mosaic virus (ATMV)	Multicellular tumor spheroids (LoVo/Adr cells)	LoVo/Adr xenograft tumor nude mouse model	^[^ ^108^ ^]^
Dendritic peptides	Membrane‐breaking nanoparticles (MBNs)	Multicellular tumor spheroids (SKOV3/R cells)	SKOV3/R xenograft tumor nude mouse model	^[^ ^109^ ^]^

Biomimetic technologies to achieve deep penetration of tumors have high skill requirements and specific experimental conditions but can produce significant results. Undoubtedly, this will be one of the key research directions in the future.

## 5 Penetration Effect Evaluation Methods

To date, in vitro models and animal experiments in vivo are mainly used to investigate the penetration effect of nanomedicines in solid tumors.

### 5.1 In Vitro Models

The development of rudimental evaluation models in vitro is crucial and imperative for the evaluation of penetration ability due to the time and money‐saving properties.^[^ ^111^ ^]^ Preliminary results can be obtained by analyzing the results of in vitro models. Nevertheless, the gap between in vitro and in vivo will be the main obstacle. In some models, counterfeit polarity, abnormal cell metabolism, and protein expression or other disadvantages may occur. Therefore, in vitro models should simulate the internal environment as much as possible, including cell–cell and cell–ECM interactions, tissue‐specific architecture, and mechanical and chemical cues.

#### 5.1.1 Multicellular Spheroids

As a rudimentary and significant method with simplicity and convenience, the multicellular spheroid method has been applied in the assessment of nanomedicine penetration in solid tumors for a long time.^[^ ^112^ ^]^ Multicellular spheroids are clusters of tumor cells with many similar properties to those of solid tumors, and can provide powerful evidence of the penetrability of nanomedicines.

**Figure 13:**
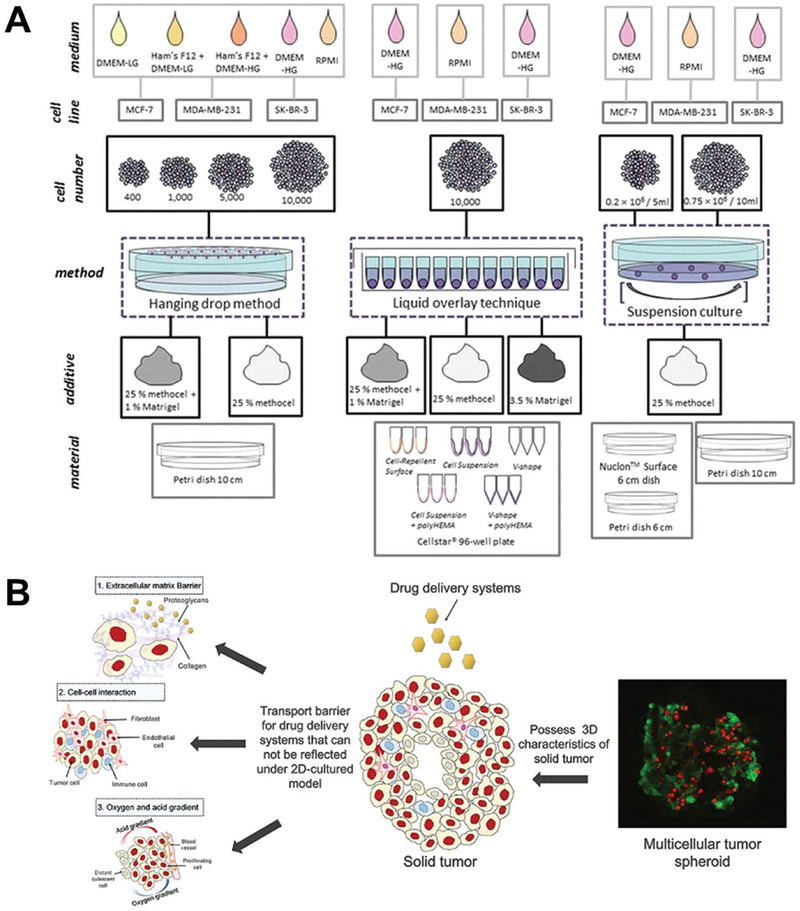
A) Preparation methods and processes of multicellular spheroids. Reproduced with permission.^[^ ^113b^ ^]^ Copyright 2016, Springer Nature. B) Application of multicellular spheroids in the evaluation of penetration effects. Reproduced with permission.^[^ ^113a^ ^]^ Copyright 2018, Elsevier B.V.

Three methods are practical for the preparation of multicellular spheroids (**Figure** **13**A).^[^ ^113^ ^]^ 1) Suspension culture: maintaining the speed of cells or culture medium to form a sphere and reducing the effect of gravity and promoting spontaneous aggregation.^[^ ^114^ ^]^ 2) Non‐adherent surface culture (liquid overlay technique): culturing cells on a non‐adherent surface to prevent attachment to the substrate and promote the formation of spheroids.^[^ ^115^ ^]^ 3) Hanging drop culture: using the surface tension and gravity of cell droplets suspended on the bottom of the cup lid, so that cells will be forced to gather into a spherical shape.^[^ ^116^ ^]^

The multicellular spheroid model recapitulates physiological cell–cell and cell–ECM interactions and allows researchers to obtain the result apart from complex factors in vivo, allowing the precise control of a single variable (Figure 13B).^[^ ^113^, ^117^ ^]^ Spheroids with diameters larger than 400–500 µm can form quiescent cell cores and proliferating cell outer layers, preserving oxygen and pH gradients in human tumor tissues.^[^ ^113a^ ^]^ However, it is difficult to simulate blood perfusion and some other dynamic characteristics in solid tumors using the multicellular spheroid model.^[^ ^113a^ ^]^ Although there has been an attempt to build vascularized 3D models of tumors in vitro, it remains a complex task to be widely available as in vitro models.^[^ ^118^ ^]^ Furthermore, limited by the strict conditions of culture, only certain cell types can be used to create multicellular spheroids.^[^ ^119^ ^]^ Additionally, given that the synthesis and analysis methods have not been standardized, the multicellular spheroid models prepared in different laboratories are quite different, and it is therefore difficult to directly compare the penetrability of different nanomedicines.^[^ ^113a^ ^]^

#### 5.1.2 Multicellular Layers

Multicellular layers, another in vitro method, were first proposed by Wilson et al.^[^ ^120^ ^]^ and can be seen used to evaluate the penetration ability of nanomedicines.^[^ ^117b^ ^]^ Generally, tumor cells are grown on collagen‐coated microporous Teflon membranes or other conditions that are suitable for the cell to grow in a layer shape. After the formation of a multicellular layer model, nanomedicine is added on one side of the multicellular layers, observing nanomedicine on the other side and evaluating the penetration ability.^[^ ^121^ ^]^

Similar to multicellular spheroids, tailor‐made multilayer cells can mimic part of the characteristics of the microenvironment and reflect some properties of solid tumors, such as gradients of nutrient concentration, which is crucial for researchers to assess the penetrability of nanomedicines.^[^ ^117b^ ^]^ However, the multilayer cell model also has issues similar to multicellular spheroids such as the difficulty to simulate some of the dynamic characteristics in vivo. Nevertheless, compared with multicellular spheroids, it is less close to human tumor tissues in morphology, cannot form oxygen and pH gradients, and is less convenient to prepare; therefore, its application scope is minimal.

### 5.2 Characterization in Animal Models

Considering the inevitable limitations, even though in vitro methods are powerful ways to study penetration, experiments in animal models cannot be replaced.

#### 5.2.1 Extraction of Animal Tissue Samples

In general, fluorescent‐labeled nanomedicines are used to observe the penetrability in animal models. After a period of injection, animals are sacrificed at a different time points and their tumors are excised to assess the depth of penetration.^[^ ^122^ ^]^ This has proven to be a very intuitive method to detect the penetration depth of nanomedicines. Similarly, on the premise that solid tumor tissues and model animals are separated in time, such a method possesses both the convenience of experiments in vitro and the accuracy of animal model experiments, which can be widely used in basic nanomedicine research. However, this method has high costs and a long experimental period. Furthermore, due to individual differences in animals, the characteristics of the excised tumors, such as morphology, size, and vascular distribution, are different and it may therefore be difficult to compare the penetration effects of different individuals.

#### 5.2.2 Penetrability Detection In Vivo

Due to the development of some fluorescent microscopy techniques, the visualization of nanomedicine in solid tumors in vivo can be detected without the need for an excising tumor. There are several mature technologies that have been applied to assess penetrability in vivo, including magnetic resonance imaging, computed tomography, and confocal laser scanning microscopy.^[^ ^123^ ^]^

Detecting the nanomedicine penetration of model animals in real‐time can be achieved through the methods above. However, this method is not widely used in basic research due to the extremely complex internal environment with a large amount of interference information. Therefore, the accuracy of the results is greatly reduced and it is difficult to compare the penetration effects between different individuals. Additionally, the operation of live animals also needs to be delicate, which increases the complexity of experiments to some extent.

## 6 Conclusions and Perspectives

The deep penetration of nanomaterials into tumors is a crucial step to achieve radical advances in the treatment of solid tumors and in the prevention of recurrence. In existing strategies for the promotion of solid tumor penetration, surface modification appears as a simple and practical engineering transformation of nanomedicines to confer them with permeability, in the case of guarantee nanomedicine original nature, such as drug‐loading capacity, photothermal performance, and microenvironment‐responsive performance. Additionally, on account of the direct contact between the surface of nanomedicine and solid tumor, the performance will work first while arriving in the tumor site, which ensures the subsequent drug release and killing tumor steps, as well as maximizes treatment effect as much as possible. This review systematically summarized the complex structure of the solid tumor microenvironment and its influence on nanomedicine penetration, discussing the existing surface modification strategies to promote penetration and the future potential applications. Furthermore, it summarized the existing commonly used permeability detection methods that will contribute to the future development of this field.

In the future, if the problem of tumor penetration can be greatly improved, the therapeutic effects of multiple tumor treatment methods will also be significantly enhanced. Chemotherapy may produce better results as chemicals are released deeper within the tumor. In the process of radiotherapy, photothermal therapy, and photodynamic therapy, with the respective in‐depth delivery of radiosensitizers, photothermal agents, and photosensitizers, the therapeutic efficiency can also be greatly improved. With regards to immunotherapy, the delivery of immune adjuvants, such as checkpoint inhibitors, also require deep tumor penetration, and the in‐depth killing of tumors will lead to the release of more antigens, resulting in a better immunotherapeutic effect.^[^ ^124^ ^]^ Furthermore, as the research on simple and feasible surface modification strategies matures and becomes widely used in the design of nanomedicines, it is likely to notably promote the clinical translation of nanomedicine. On the one hand, the penetration problem of tumors is one of the main shortcomings restricting the good therapeutic effect of nanomedicine in the human body, so the development of nanomedicine with good penetration effect in the human body is expected to bring nanomedicine up to the standard of clinical application from the perspective of therapeutic effect. On the other hand, the achievement of clinical translation requires the large‐scale production of nanomedicines; therefore, well‐developed and simple surface modification strategies will increase the feasibility of achieving this goal.

However, for now, despite the excellent results produced in this field, there remain challenges that need to be overcome prior to the application of these techniques. First, the design of some nanomedicine surface modifications for the promotion of tumor penetration remains too complex, reducing the preparation efficiency without further improvements of the therapeutic effect. Similarly, there are issues related to the quantitative production and clinical translation of nanomedicines, which is inconsistent with the original intention of surface modification applied in the field of drug delivery. Second, there is no unified standard for the evaluation of penetration experiments, leading to major differences in the experimental methods, in vitro models, and animal models used in the various laboratories, and thus no direct comparison can be made between the penetration promotion effects produced by different surface modification strategies. Finally, given the large difference between solid tumors in animals and human, the good permeability effect obtained in animal models, such as in mice, cannot directly represent the effect in humans. Therefore, it is necessary to consider the use of multiple animal models or the establishment of tumor models in vivo that are more similar to human solid tumors for the evaluation of penetration.

Thus, the promotion of deep tumor penetration of nanomedicines through surface modification is still in the initial stages, with various ideas and studies that can be further implemented and completed. We hope that the summary of this review can give researchers more inspiration for work in nanomedicine surface modification and to further promote the development of this work.

## Conflict of Interest

The authors declare no conflict of interest.
